# Drinking Over the Lifespan: Focus on Older Adults

**DOI:** 10.35946/arcr.v38.1.14

**Published:** 2016

**Authors:** Kristen L. Barry, Frederic C. Blow

**Affiliations:** Kristen L. Barry, Ph.D., is a research professor and Frederic C. Blow, Ph.D., is a professor, both in the Department of Psychiatry at the University of Michigan, Ann Arbor, Michigan

**Keywords:** Alcohol consumption, alcohol use and dependence, alcohol misuse, older adult, elderly, special populations, treatment, intervention, screening

## Abstract

A substantial and growing number of older adults misuse alcohol. The emerging literature on the “Baby Boom” cohort, which is now reaching older adulthood, indicates that they are continuing to use alcohol at a higher rate than previous older generations. The development and refinement of techniques to address these problems and provide early intervention services will be crucial to meeting the needs of this growing population. This review provides background on the extent of alcohol misuse among older adults, including the Baby Boom cohort that has reached age 65, the consequences of misuse, physiological changes related to alcohol use, guidelines for alcohol use, methods for screening and early interventions, and treatment outcomes.

In 2010, when the leading edge of the post–World War II “Baby Boom” reached age 65, the United States began a period of increased growth in its older adult population. By 2030, it is expected that there will be 72.1 million adults age 65 or older living in the United States, almost double the 2008 population. Those older adults will represent 19.3 percent of the U.S. population, compared with 12.9 percent of the population in 2009 (Administration on Aging 2011; U.S. Census Bureau 2013). The United States is facing a “silver tsunami” that will greatly influence many segments of society, including the economy, large-scale societal programs, and the health care system. Aging research focused on “older adulthood” defines this cohort in a variety of ways, most commonly as age 50 or older, 60 or older, and 65 or older (Substance Abuse and Mental Health Service Administration [SAMHSA] 2012*a*). In this article, “older adulthood” refers to individuals who are age 65 or older, unless otherwise indicated in the text (Bartels and Naslund 2013).

The aging of the Baby Boom population will severely tax the current health care system (Bartels and Naslund 2013). There is a paucity of clinicians specializing in geriatric medicine and geriatric psychiatry. In addition, there is no single agency in the United States in charge of the mental and physical health care of this vulnerable and growing population of older adults who are more likely than previous older generations to experience problems related to mental health and alcohol use. In fact, as the Baby Boom cohort moves into older adulthood, they are likely to use more alcohol than previous generations of older adults. Misuse and abuse of alcohol, and the combination of alcohol with the use of some medications (including benzodiazepines, sedatives, and opioid analgesics), can lead to negative health outcomes. With the size of the emerging older population and their comparatively higher acceptance of alcohol and drugs, there is a growing concern that there will be a substantial increase in the number of older adults at risk for alcohol misuse and abuse (Agency for Healthcare Research and Quality 2010; Korper and Council 2002).

Increased at-risk use and abuse of alcohol in older adults will present unique challenges in terms of recognition, interventions, and determining the most appropriate treatment options, when needed. Alcohol problems in this age group often are not recognized and, if recognized, generally are undertreated. However, older adults are more likely than younger adults to seek services from their primary and specialty care providers, which opens the door to greater recognition and assistance for those who drink above guidelines. Health care providers who work with older adults have a unique opportunity to observe and treat the repercussions of alcohol misuse, abuse, and dependence.

This review focuses on the prevalence of alcohol misuse, abuse, and dependence in older adults; guidelines for use; physiological changes in sensitivity and tolerance; and the efficacy and effectiveness of screening, interventions, and treatments in this age group.

## Prevalence of Alcohol Misuse Among Older Adults

### At-Risk Alcohol and Drug Use

Over the last three decades, studies have estimated that the prevalence of at-risk and problem drinking among older adults ranges from 1 percent to 16 percent (Menninger 2002; Moore et al. 1999; SAMHSA 2004, 2007). These rates vary widely, depending on the definitions of older adults, at-risk and problem drinking, alcohol abuse/dependence, and the methodology used in obtaining samples. The 2013 National Survey on Drug Use and Health (NSDUH), for example, found heavy drinking (defined as drinking 5 or more drinks on the same occasion on each of 5 or more days in the past 30 days) among 5.6 percent of 50- to 54-year-olds, 3.9 percent of 55- to 59-year-olds, 4.7 percent of 60- to 64-year-olds, and 2.1 percent of those over age 65 (SAMHSA 2014). It found even higher rates of binge drinking (defined as drinking 5 or more drinks on the same occasion on at least 1 day in the past 30 days) among these age groups; 23.0 percent of 50- to 54-year-olds, 15.9 percent of 55- to 59-year-olds, 14.1 percent of 60- to 64-year-olds, and 9.1 percent of those over 65 (SAMHSA 2014).

In addition, studies find higher rates of at-risk use and abuse among people seeking health care, because people with alcohol dependence who have at-risk use and/or are beginning to experience consequences related to that use are more likely to seek medical care (Oslin 2004). Early studies in primary care settings found that 10 to 15 percent of older patients met criteria for at-risk or problem drinking (Barry 1999), defined by the National Institute on Alcohol Abuse and Alcoholism (NIAAA) as more than one-half to 1 drink per day or 7 drinks per week for women and more than 2 drinks per day or 14 drinks per week for men (Gunzerath et al. 2004; NIAAA 1995, 2007; Willenbring et al. 2009). Because patients with a previous history of problems with alcohol or other drugs are at risk for relapse, establishing a history of use can provide important clues for future problems.

## Alcohol Misuse/Dependence

The rates of alcohol misuse/dependence in older adults are by far smaller than the rates of at-risk use. In 2002, over 616,000 adults age 55 and older reported meeting the criteria for alcohol dependence in the past year, as defined by the *Diagnostic and Statistical Manual of Mental Disorders, Fourth Edition* (DSM–IV). They represented 1.8 percent of those age 55–59, 1.5 percent of those age 60–64, and 0.5 percent of those age 65 or older (SAMHSA 2002). Later data from NSDUH showed that 780,000 older adults had alcohol abuse/dependence (SAMHSA 2012*a*). The NSDUH also has shown that illicit drug use increased from 1 percent in 2003 and 2004 to 3.9 percent in 2013 (SAMHSA 2014), suggesting that both alcohol and drug use have increased slightly in older adulthood.

Although substance dependence is less common in older adults when compared with younger adults, the mental and physical health consequences in this age group are serious (Barry and Blow 2010). Indeed, although the majority of older adults who are experiencing drinking-related problems do not meet criteria from DSM–IV for alcohol abuse or dependence (SAMHSA 2012*b*), the diagnostic criteria that relate to the physical and emotional consequences of alcohol use may be especially important in identifying alcohol use disorders in older adults.

## Impact of Physiological Changes on Alcohol Consumption

Older adults are more vulnerable to the physiological effects of alcohol than younger adults (Gargiulo et al. 2013). Alcohol consumption in amounts considered light or moderate for younger adults may have untoward health effects in older people because it is processed differently (Ferreira and Weems 2008; Gargiulo et al. 2013). In particular, as people age, liver enzymes that metabolize alcohol and other drugs are less efficient, and the central nervous system becomes more sensitive to drugs. In addition, age-related decreases in lean body mass result in a decrease in the aqueous volume of cells, which in turn increases the effective concentration of alcohol and other mood-altering chemicals in the body. Because drinking comparable amounts of alcohol produces higher and longer-lasting blood alcohol levels in older adults than in younger people, many problems common among older people, such as chronic illness and poor nutrition may be exacerbated by even small amounts of alcohol. Likewise, because older adults who drink are more likely to take alcohol-interactive medications than younger drinkers (Breslow et al. 2015), they may be at increased risk for adverse alcohol-medication interactions. Clinicians who treat older patients can assess the number of drinks per day, the number of drinking days, and any binge drinking to begin to address the health implications of an individual’s pattern of use.

## Risks of Heavier Drinking in Older Adulthood

Studies analyzing data from the National Health and Retirement Study (Bobo et al. 2013) found that, although overall alcohol consumption declined with age, for a minority of individuals, consumption increased. Those who increased their consumption over time were more likely to be affluent, highly educated, male, Caucasian, unmarried, less religious, and perceive themselves to be in excellent health.

Heavy drinking or binge drinking is of particular concern in all age groups. But, as people age, binge drinking is thought to pose even higher risks for morbidity, including accidents, and mortality. To evaluate the relationship between drinking patterns and health in older adults, Holahan and colleagues (2012) studied 446 people with a mean age of 62 at the beginning of the study. Study participants were “moderate drinkers” based on NIAAA’s guidelines of drinking at least half a drink per day but no more than half a drink per day for women and two drinks per day for men (NIAAA 2007). Some also were moderate drinkers who had periods of episodic heavy drinking or binge drinking defined as drinking four or more drinks for women and five or more drinks for men on the occasion of the largest amount of drinking. Overall, the study found that moderate drinkers who engaged in episodic heavy drinking were more than twice as likely to die within 20 years compared with regular moderate drinkers.

Older adults also are at greater risk for harmful drug interactions, injury, depression, memory problems, liver disease, cognitive changes, sleep problems, cancer, and diabetes that can be related to heavier alcohol consumption (Blow and Barry 2012; Holahan et al. 2012; Moore et al. 2007; Mukamal et al. 2010; Wu and Blazer 2011). In addition, heavier drinking in this age group can significantly affect a number of other conditions in older adults (Fleming and Barry 1992), including mood disorders, sleep, and pain, as well as general health functioning (American Medical Association [AMA] 2008; Blow et al. 2002).

Not all research finds negative consequences of alcohol for older adults. In particular, one study suggests alcohol may decrease the risk of coronary heart disease. The Coronary Heart Disease Study looked at factors, including alcohol consumption, related to the risk of coronary heart disease in older adults. In the older adult sample of more than 4,400 subjects who were free of known coronary heart disease at baseline, consumption of 14 or more drinks per week was associated with the lowest risk of coronary heart disease in the long term (Mukamal et al. 2010). Because of the risks of other disorders related to heavy alcohol use, these findings need to be placed in the context of known adverse effects of heavy drinking and the established recommended guidelines for alcohol use in older adults.

In addition to concerns regarding the misuse of alcohol alone, up to 19 percent of older Americans combine alcohol and medications in a way that can be considered misuse (NIAAA 1998*a*). Mixing alcohol and psychoactive medications such as benzodiazepines, sedatives, and opioid analgesics has the potential for very serious negative outcomes that prescribing physicians should discuss with older adult patients. And although the use and misuse of illicit drugs is less common in the current cohort of older adults—1.8 percent among people age 50 and over in 2002–2003 (SAMHSA 2007)—research suggests that that number is likely to increase as a result of the aging of the Baby Boom generation (Bartels and Naslund 2013).

## Screening and Brief Interventions

Alcohol screening and brief interventions offer opportunities for early detection, focused motivational enhancement, and targeted encouragement to seek needed substance abuse treatment, where appropriate. The majority of older adults who misuse alcohol do not need formal specialized substance abuse treatment. Rather, many can benefit from screening and brief interventions regarding their drinking (Kuerbis et al. 2015; Pilowsky and Wu 2012).

### Screening

Screening, including rapid prescreening, for alcohol use and potential deleterious consequences of alcohol use is a critical first step in identifying individuals who may need additional in-depth assessment and those who may benefit from brief interventions and/or treatment. Screening generally identifies at-risk and harmful substance use. In contrast, more extensive assessments measure the severity of the substance use; problems and consequences associated with use; factors that may be contributing to substance abuse; and other characteristics of the problem. The screening and assessment process should help clinicians determine if the level of alcohol use is appropriate for a brief intervention or if it warrants a different approach. Clinicians can administer simple questions about alcohol use via a paper-and-pencil or computerized questionnaire or as part of a clinical interview and can follow up with additional questions as needed (adapted from *Helping Patients Who Drink Too Much: A Clinician’s Guide*, NIAAA 2007).

The Alcohol Use Disorders Identification Test (AUDIT) is a useful validated brief screening instrument for excessive drinking developed by the World Health Organization (WHO) (Barry and Fleming 1993; Fiellin et al. 2000; Schmidt et al. 1995). A 10-item questionnaire that collects alcohol-related information about the previous year only, AUDIT often is used without a clinical examination. And although the recommended cut-off score for the AUDIT typically is 8, early research suggests that, for older adults, a score of 5 should trigger additional clarifying questions (Barry et al. 2001).

Researchers at the University of Michigan have developed the Michigan Alcoholism Screening Test—Geriatric Version (MAST-G) and the shorter version, the Short Michigan Alcoholism Screening Test—Geriatric Version SMAST-G (Blow et al. 1992) as a screening instrument specifically for use with older adults in a variety of settings. The MAST-G includes items unique to older problem drinkers and relies on a 24-item scale with good sensitivity and specificity in older adults. The SMAST-G, with 10 items, is a validated shortened form of the MAST-G.

### Brief Interventions

There is a large body of evidence showing that brief interventions delivered in a variety of health care and social service settings can effectively reduce drinking, particularly for at-risk and problem users under age 60. Indeed, researchers have conducted over 100 studies of brief intervention techniques over the past 25 years. The general format of brief alcohol interventions has been relatively consistent over time (Barry 1999). Typically, interventions include personalized feedback based on a person’s responses to screening questions and generic messages to cut down on or stop drinking. In primary care, brief interventions for a patient with at-risk or problem drinking might include a few simple, straightforward comments about concerns regarding the patient’s pattern of alcohol use and recommendations that the patient reduce or stop drinking or might include several short counseling sessions followed by telephone followup (NIAAA 2007). Brief interventions use motivational interviewing principles and nonjudgmental language, eliciting the potential to change and/or consider change.

Early studies in Europe and other countries demonstrated 10 to 20 percent reductions in drinking for people receiving a brief intervention compared with people in control groups (e.g., Saunders et al. 1993). In addition, meta-analyses of randomized controlled studies examining the effectiveness of brief interventions find that these techniques generally reduce drinking in the intervention group. For example, Whitlock and colleagues (2004) examined 39 studies. Among the 12 that met their criteria for inclusion, participants reduced their average number of drinks per week by 13 to 34 more than did control subjects. The majority of brief intervention trials have been conducted in primary care settings (e.g., Fleming et al. 1997; Whitlock et al. 2004). However, a number of successful brief alcohol intervention trials have been conducted in emergency settings with individuals of varying ages and levels of alcohol use (see Havard et al. 2008 and Carey et al. 2012 for meta-analyses).

The few studies of brief interventions with older adults have found them to be effective in reducing at-risk alcohol use (e.g., Fleming et al. 1997; Moore et al. 2011). Specifically, screening and brief interventions in a variety of health care and social service settings have reduced alcohol consumption among older adults, with these reductions sustained for 2 to 18 months (Fleming et al. 1997; Moore et al. 2011; Schonfeld et al. 2010).

## Alcohol Treatment

Although alcohol abuse/dependence is a significant and growing health problem for the increasing population of older adults in the United States (AMA 1996; Bartels and Naslund 2013), there have been very few systematic studies of alcohol treatment outcomes for older adults (Bobo et al. 2013; Oslin et al. 2005; Satre et al. 2012). Because traditional substance use treatment programs have provided services to few older adults, sample size issues have been a barrier to studying treatment outcomes for older adults who meet criteria for abuse/dependence.

The few studies in this area generally have focused on the completion of prescribed treatment activities and adherence to drinking goals—generally abstinence. Of note is that older adults with alcohol use disorder were significantly more likely to complete treatment than younger adults. More recently, Lemke and Moos (2003) found that older adults in residential treatment had better long-term outcomes than matched groups of young and middle-aged patients. Longer duration of care and more use of self-help groups positively influenced outcomes. They also found that older adults were more likely to complete treatment and had more days of sobriety than younger adults. Satre and colleagues (2012) compared 5-year treatment outcomes for adults age 55 or older, age 18–39, and age 40–54. They found that the 55-and-over group were less likely to be alcohol/drug dependent at treatment entry and stayed longer in treatment. They also were less likely, at 5 years posttreatment, to have family and friends that encouraged alcohol use. Older females were more likely than any other group to be abstinent at followup.

Age of onset of alcohol problems has been posited as a consideration in treatment outcomes. Specifically, some researchers have hypothesized that people who experience alcohol problems later in life are more likely to have better outcomes than those whose alcohol problems start earlier. A small study by Schonfeld and Dupree (1991) used a matched-pairs, post hoc design to determine rates of completion of a 6-month day-treatment program. They compared alcohol-dependent male and female patients, age 55 or older, whose alcohol problems began before age 50 (early onset) with those who began problem drinking after age 50 (late onset). The late-onset group was significantly more likely to complete treatment.

There remain major limitations in the treatment literature for older adults, including insufficient drinking outcome data, failure to report on treatment dropouts, and variations in definitions of treatment completion. However, with the aging of the Baby Boom cohort and the potential for treatment needs in a larger population of older drinkers, there is beginning to be a greater emphasis on determining treatment outcomes for this population.

## Conclusions

The health care delivery system remains one of the most efficient and effective venues in which to detect at-risk drinking, combined use of alcohol and psychoactive prescription medications, and comorbid mental and physical health conditions in older adults. It also is a key setting for interventions to improve the quality of life for many older adults. And there is a growing body of knowledge about useful screening tools, easy-to-use intervention methods, and brief and long-term treatments for use in the health care setting with this age group.

However, clinicians may need brief skills training to be able to assess effectively and more rapidly the quantity and frequency of alcohol use as well as any comorbid physical and mental health issues (e.g., depression and suicide risk) in this age group. Health care delivery occurs in an increasingly fast-paced environment, with many competing demands being placed on providers. Therefore, targeted training that focuses on screening and nonjudgmental intervention techniques with older adults could improve both skills and efficiency. Changes in the health care environment in the United States underscore the importance of using these brief cost-effective techniques with older adults with substance-related problems. Because many older adults confront multiple challenges, including social isolation, loss and grief, economic difficulties, and physical illnesses, health care professionals may assume that all symptoms are an inevitable result of these difficulties and may not recognize characteristics of at-risk drinking or alcohol abuse. The use of screening and intervention techniques that take into account issues specific to older adults moves the field toward providing best practice care to a potentially vulnerable population.
